# OX40 and LAG3 are associated with better prognosis in advanced gastric cancer patients treated with anti-programmed death-1 antibody

**DOI:** 10.1038/s41416-020-0810-1

**Published:** 2020-03-23

**Authors:** Hirofumi Ohmura, Kyoko Yamaguchi, Fumiyasu Hanamura, Mamoru Ito, Akitaka Makiyama, Keita Uchino, Hozumi Shimokawa, Shingo Tamura, Taito Esaki, Kenji Mitsugi, Yoshihiro Shibata, Hisanobu Oda, Kenji Tsuchihashi, Hiroshi Ariyama, Hitoshi Kusaba, Yoshinao Oda, Koichi Akashi, Eishi Baba

**Affiliations:** 1 Department of Medicine and Biosystemic Science, Kyushu University Graduate School of Medical Sciences, Higashi-ku, Fukuoka, Japan; 2 Department of Hematology/Oncology, Japan Community Healthcare Organization Kyushu Hospital, Fukuoka, Japan; 3 Department of Clinical Oncology, NTT Medical Center Tokyo, Tokyo, Japan; 4 Department of Medical Oncology, National Hospital Organization Kyushu Medical Center, Fukuoka, Japan; 5 Department of Gastrointestinal and Medical Oncology, National Kyushu Cancer Center, Fukuoka, Japan; 6 Department of Medical Oncology, Hamanomachi Hospital, Fukuoka, Japan; 7 Department of Medical Oncology, Fukuoka Wajiro Hospital, Fukuoka, Japan; 8 Department of Medical Oncology, Saiseikai Fukuoka General Hospital, Fukuoka, Japan; 9 Department of Medicine and Comprehensive Biosystemic Science Faculty, Kyushu University, Fukuoka, Japan; 10 Department of Anatomic Pathology, Graduate School of Medical Sciences, Kyushu University, Fukuoka, Japan; 11 Department of Oncology and Social Medicine, Kyushu University Graduate School of Medical Sciences, Fukuoka, Japan

**Keywords:** Prognostic markers, Gastric cancer

## Abstract

**Background:**

Anti-PD-1 monoclonal antibody, nivolumab, has shown efficacy for advanced gastric cancer (AGC). However, the specific immune cell subsets predominantly activated during the period of anti-PD-1 therapy for AGC have not been clarified.

**Methods:**

Peripheral blood of 30 AGC patients treated with nivolumab was prospectively obtained before the initial and second administrations and at the time of progressive disease (PD). The proportions of immune cell subsets and the serum concentrations of cytokines were systematically analysed by flow cytometry. Associations of subsets and serum cytokines with therapeutic effects were evaluated.

**Results:**

After the initial administration, significant increases in activated central/effector memory, activated effector T cells, and activated T-helper 1 subsets were observed. At the time of PD, activated regulatory T cells, LAG3-positive CD4+/CD8+ T cells, and TIM3-positive CD4+/CD8+ T cells increased significantly. Significant positive correlations were shown between progression-free survival and proportions of LAG3-positive CD4+/CD8+ T cells and of OX40-positive CD4+/CD8+ T cells (log-rank *p* = 0.0008, 0.0003, 0.0035 and 0.0040).

**Conclusions:**

Nivolumab therapy enhances activation of central/effector memory and effector subsets of CD4+/CD8+ T cells. The expression levels of LAG-3 and OX40 on T cells correlated with the efficacy of nivolumab therapy and could be reasonable biomarkers for anti-PD-1 therapy.

## Background

Gastric cancer is one of the most common malignancies and is the third leading cause of cancer-related death in the world.^1^ For patients with advanced and recurrent disease, platinum and fluoropyrimidine-based systemic chemotherapy is recommended as first-line treatment.^2^ For patients with disease refractory to first-line treatment, the following agents are selected for second-line treatment: paclitaxel, docetaxel, irinotecan, anti-vascular endothelial growth factor receptor 2 (VEGFR-2) antibody ramucirumab monotherapy, or ramucirumab plus paclitaxel.^3–6^ Nivolumab, a fully human anti-programmed death-1 (PD-1) monoclonal antibody, demonstrated a significant survival benefit for advanced or metastatic gastric cancer (AGC) previously treated with two or more chemotherapy regimens.^7^ PD-1 is the type I membrane glycoprotein expressed on the surface of T cells, B cells, and natural killer (NK) cells.^8,9^ The expression of PD-1 on T cells is promoted by T cell-activation induced via antigen presentation. Under the condition of continuous T cell-activation, including chronic infection or malignancies, PD-1 is strongly expressed on exhausted T cells. Exhausted T cells are characterised by the loss of function of cytokine production or cytotoxic activity.^10^ T cells receive an inhibitory signal after binding of PD-1 and the ligands PD-L1 or PD-L2, expressed on antigen-presenting cells (APCs) and tumour cells, resulting in the suppression of proliferation, cytokine production, and cytotoxic activity. Anti-PD-1 antibody is thought to activate tumour-specific T cells by interfering with the ligation of PD-1 on tumour-specific T cells and PD-L1/L2 on tumour cells in both priming and effector phases.^11^ Thus, it has been suggested that anti-PD-1 therapy modulates systemic host-immune reactions and exerts an anti-tumour effect. In fact, human anti-PD-1 antibody has shown efficacy in the treatment of malignancies derived from various organs, including malignant melanoma, non-small cell lung cancer, renal cell carcinoma, Hodgkin’s lymphoma, head and neck cancer, and gastric cancer.^12–17^ However, the median overall survival time of AGC patients who received anti-PD-1 therapy in the previous study was around 5 months, and their prognosis remains poor.

Anti-PD-1 antibody is thought to activate tumour-specific T cells by interfering with the ligation of PD-1 expressed on tumour-specific T cells and PD-L1/L2 on tumour cells. Thus, PD-1-expressing T cells in the tumour site have been thought to be the main target of PD-1 blockade.^18^ In addition, PD-1-expressing T cells are observed not only in secondary lymphoid organs, but also in peripheral blood. T cells are activated through antigen presentation by APCs, and they then express PD-1.^19^ Therefore, anti-PD-1 therapy may contribute to anti-tumour effects both directly and indirectly by inhibiting regulatory signalling in not only PD-1-expressing T cells in the tumour site, but also in PD-1-expressing T cells in the secondary lymphoid organs and peripheral blood. Patients who received anti-PD-1 therapy were divided into two groups: those who obtained a therapeutic effect, and they consist of the tail-plateau part in the Kaplan–Meier curve; and those who showed a poor therapeutic effect. Moreover, the effects of anti-PD-1 therapy on T cell subsets other than T cells in the tumour site and the subsequent impact on the systemic immune system have not yet been elucidated in AGC patients. Thus, with the peripheral blood obtained from patients before and after anti-PD-1 therapy, systemic immune cell subsets playing a main role in the anti-tumour effect and ones changing in responders or poor responders were examined.

In the present study, the specific subsets of immune cells predominantly activated and the changes in serum cytokine levels during the period of anti-PD-1 therapy for AGC were analysed, referencing standardised assays from the Human Immunology Project^20^ and our previous study of anti-PD-1 therapy for malignant melanoma.^21^ In addition to these studies, the expressions of exhaustion markers and costimulatory molecules were evaluated. Some of these molecules have recently been the targets of immunotherapy, and several clinical trials are ongoing. These molecules were also evaluated in order to identify cell populations that have the potential to show anti-tumour effects with anti-PD-1 therapy. Based on our previous small cohort study,^22^ more eligible cases were registered, and analysis of immune cell subsets, quantification of cytokine and immunohistological study were performed in this study.

## Methods

### Patients

A total of 30 patients with AGC who were scheduled to receive the anti-PD-1 antibody nivolumab from November 2017 to December 2018 at participating institutions were registered. Eligible patients were aged 20 years or older and had histologically confirmed adenocarcinoma of the stomach or gastro-oesophageal junction that was metastatic or locally advanced but unresectable. Nivolumab (3 mg/kg) was administered intravenously every 2 weeks, and it was continued until PD by investigator assessment, intolerable adverse events, refusal by the patient, or investigator decision to withdraw treatment. Tumour responses were assessed by the investigator by physical examination, diagnostic imaging (computed tomography or magnetic resonance imaging), and gastrointestinal endoscopy, according to the response evaluation criteria in solid tumours (RECIST) version 1.1.^23^ CT and other diagnostic imaging were performed by the attending physician based on the clinical practice; thus, the CT evaluation period was not clearly defined. Adverse events were assessed by the National Cancer Institute Common Terminology Criteria for Adverse Events version 4.0.^24^ The medical information of each patient, including age, sex, Eastern Cooperative Oncology Group (ECOG) performance status (PS), human epidermal growth factor receptor 2 (HER2) status, previous systemic treatments, total cycles of anti-PD-1 therapy, and survival time, was examined using electronic medical records. HER2 status was defined as positive (immunohistochemistry score 3+, or immunohistochemistry score 2+ and fluorescence in situ hybridisation-positive) based on the staining patterns of surgical or biopsy specimens.

This study was approved by the ethics committees of all participating institutions and was performed according to the guidelines for biomedical research specified in the Declaration of Helsinki. Written, informed consent was obtained from each patient participating in this study.

### Peripheral blood immune cells and serum

Peripheral blood was collected using acid citrate dextrose solution-added blood collection tubes (8.5 mL) and serum-separating tubes (8 mL) from each patient before the initial and second administrations of nivolumab and at the time of PD. Peripheral blood mononuclear cells (PBMCs) were separated by gradient centrifugation of acid citrate dextrose solution-added blood collection tubes with Ficoll (Ficoll-Paque PLUS, GE Healthcare, Little Chalfont, UK), washed with PBS containing 2% FBS and 1 mM EDTA (FACS buffer), and then the cells were resuspended in FACS buffer on ice for subsequent flow cytometry. Otherwise, separated PBMSs were cryopreserved at −80 °C. Whole blood in serum-separating tubes was left to allow clotting at room temperature for 30 min. Clot was then removed by centrifuging at 1000*×g* for 10 min at 4 °C to separate serum.

### Flow cytometry

As described previously,^21^ a total of 5 × 10^5^ PBMCs resuspended in 50 μL FACS buffer were incubated with fluorophore-conjugated antibodies at a final concentration of 1–5 μg/mL in the dark for 20 min on ice. The cells were then washed twice with FACS buffer, resuspended in 200 μL FACS buffer, and analysed. Flow cytometry was performed using FACSAria III (BD Bioscience, Tokyo, Japan). The data of flow cytometry were exported as FCS files and analysed with FlowJo version 9 (Tomy Digital Biology, Tokyo, Japan). The 10 panels of fluorophore-conjugated monoclonal antibodies for immunophenotyping are listed as follows: panel 1 (for the detection of naïve/memory/effector T cells and activated T cell phenotypes), FITC-CCR7/CD197 (G043H7, BioLegend, San Diego, CA, USA), PE-CD38 (HB-7, BioLegend), PE-Cy7-CD3 (HIT3a, BioLegend), APC-CD8 (SK1, BioLegend), APC-Cy7-CD45RA (HI100, BioLegend), BV421-HLA-DR (L243, BioLegend) and BV510-CD4 (OKT4, BioLegend); panel 2 (for the detection of regulatory T cells; Tregs), FITC-CD45RO (UCHL1, BioLegend), PE-CD127 (A019D5, BioLegend), PerCP-Cy5.5-CD8 (SK1, BioLegend), PerCP-Cy5.5-CD14 (63D3, BioLegend), PE-Cy7-CCR4/CD194 (L291H4, BioLegend), APC-CD25 (BC96, BioLegend), BV421-HLA-DR (L243, BioLegend), APC-Cy7-CD3 (OKT3, BioLegend), and BV510-CD4 (OKT4, BioLegend); panel 3 (for the detection of activated phenotypes of Th and Tfh cells), FITC-CD3 (HIT3a, BioLegend), PE-CD38 (HB-7, BioLegend), PE-Cy7-CCR6/CD196 (G034E3, BioLegend), APC-CXCR3/CD183 (G025H7, BioLegend), APC-Cy7-CD8 (SK1, BioLegend), BV421-HLA-DR (L243, BioLegend), and BV510-CD4 (A161A1, BioLegend); panel 4 (for the detection of B cells), FITC-IgD (IA6-2, BioLegend), PE-CD24 (ML5, BioLegend), PerCP-Cy5.5-CD14 (63D3, BioLegend), PE-Cy7-CD20 (2H7, BioLegend), APC-CD27 (M-T271, BioLegend), APC-Cy7-CD3 (HIT3a, BioLegend), BV421-CD19 (HIB19, BioLegend), and BV510-CD38 (HB-7, BioLegend); panel 5 (for the detection of NK cells, dendritic cells (DCs), and monocytes), FITC-CD11c (3.9, BioLegend), PE-HLA-DR (L243, BioLegend), PerCP-Cy5.5-CD3 (HIT3a, BioLegend), PE-Cy7-CD123 (6H6, BioLegend), APC-CD19 (HIB19, BioLegend), APC-Cy7-CD16 (3G8, BioLegend), BV421-CD56 (NCAM16.2, BD), and BV510-CD14 (63D3, BioLegend); panel 6 (for the detection of Th cells and Tfh cells), FITC-CCR7/CD197 (G043H7, BD BioLegend), PE-PD1/CD279 (EH12.2H7, BioLegend), PerCP-Cy5.5-CD14 (63D3, BioLegend), PerCP-Cy5.5-CD8 (SK1, BioLegend), PE-Cy7-CCR6/CD196 (G034E3, BioLegend), APC-CXCR3/ CD183 (G025H7, BioLegend), APC-Cy7-CD45RA (HI100, BioLegend), BV421-CXCR5/CD185 (J252D4, BioLegend), and BV510-CD4 (OKT4, BioLegend); panel 7 (for the detection of costimulatory and coinhibitory markers on T cells), FITC-CD8a (RPA-T8, BioLegend), PE-ICOS/CD278 (C398.4A, BioLegend), Alexa Fluor 647-OX40/CD134 (Ber-ACT35, BioLegend), APC-Cy7-CD3 (OKT3, BioLegend), BV421-CD152/CTLA-4 (BNI3, BioLegend), and BV510-CD4 (OKT4, BioLegend); panel 8 (for the detection of exhaustion markers, PD-1 and TIM-3), FITC-CD45RA (HI100, BioLegend), PE-TIM-3/CD366 (344801, R&D Systems, Minneapolis, MN, USA), PerCP-Cy5.5-CD8 (SK1, BioLegend), PE-Cy7-PD-1/CD279 (EH12.2H7, BioLegend), APC-CCR7/CD197 (G043H7, BioLegend), BV421-CD8a (RPA-T8, BioLegend), APC-Cy7-CD3 (OKT3, BioLegend), and BV510-CD4 (OKT4, BioLegend); panel 9 (for the detection of LAG-3 and TIGIT on T cells), FITC-CD8a (RPA-T8, BioLegend), Alexa Fluor 647-LAG-3/CD223 (11C3C65, BioLegend), APC-Cy7-CD3 (OKT3, BioLegend), BV421-TIGIT (A15153G, BioLegend), and BV510-CD4 (OKT4, BioLegend); and panel 10 (for the phenotyping of OX40 and LAG3+ T cells), FITC-CCR7/CD197 (G043H7, BioLegend), APC-CD25 (BC96, BioLegend), PE-Cy7-CCR4/CD194 (L291H4, BioLegend), Alexa Fluor 647-LAG-3/CD223 (11C3C65, BioLegend), APC-Cy7-CD45RA (HI100, BioLegend), BV421-OX40/CD134 (Ber-ACT35, BioLegend), BV510-CD4 (OKT4, BioLegend), BV605-CD127 (A019D5, BioLegend), BV650-CD3 (OKT3, BioLegend) and BV785-CD8a (RPA-T8, BioLegend). Appropriate isotype control antibodies as follows were used to determine the level of background staining: FITC Mouse IgG1, κ Isotype Ctrl Antibody (MOPC-21, BioLegend), PE Mouse IgG1, κ Isotype Ctrl Antibody (MOPC-21, BioLegend), PE Rat IgG2a, κ Isotype Ctrl Antibody (RTK2758, BioLegend), PE/Cy7 Mouse IgG1, κ Isotype Ctrl Antibody (MOPC-21, BioLegend), APC Mouse IgG1, κ Isotype Ctrl Antibody (MOPC-21, BioLegend), APC/Cy7 Mouse IgG1, κ Isotype Ctrl Antibody (MOPC-21, BioLegend), Brilliant Violet 421 Mouse IgG1, κ Isotype Ctrl Antibody (MOPC-21, BioLegend), Brilliant Violet 510 Mouse IgG1, κ Isotype Ctrl Antibody (MOPC-21, BioLegend), Brilliant Violet 605 Mouse IgG1, κ Isotype Ctrl Antibody (MOPC-21, BioLegend), Brilliant Violet 650 Mouse IgG1, κ Isotype Ctrl Antibody (MOPC-21, BioLegend), Brilliant Violet 711 Mouse IgG1, κ Isotype Ctrl Antibody (MOPC-21, BioLegend) and Alexa Fluor 647 Mouse IgG1, κ Isotype Ctrl Antibody (MOPC-21, BioLegend).

### Quantification of cytokine concentrations

Serum cytokine concentrations before the initial and second administrations of nivolumab and at the time of PD were quantified with a cytometric bead assay using LEGENDplex Human Th Panel (13-plex; BioLegend) according to the manufacturer’s recommendations and analysed using the FACSAria III. The data of flow cytometry were exported as FCS files and analysed with BioLegend LEGENDplex software version 8 (BioLegend). The following cytokines were analysed: Th1 cytokines (interleukin (IL) -2, interferon gamma (IFNγ) and tumour necrosis factor alpha (TNFα)); Th2 cytokines (IL-4, IL-5, IL-6, IL-10, IL-13); Th17 cytokines (IL-17A, IL-17F, IL-21, IL-22); and Th9 cytokine (IL-9).

### Immunohistochemistry

Formalin-fixed paraffin-embedded (FFPE) tumour samples obtained before chemotherapy were available in 29 of the 30 cases. PD-Ll staining of each case was performed using FFPE tumour samples retrospectively with an anti-PD-L1 antibody (28-8, Abcam, Cambridge, UK, 1:100 dilution). FFPE samples were sliced to 3–5 μm, and slides were boiled in a microwave oven for antigen retrieval. The endogenous peroxidase was inactivated by incubating the slides with 3% hydrogen peroxide, and non-specific background staining was blocked with Protein Block Serum-Free Ready-to-use (X0909, DAKO, Glostrup, Denmark). Primary antibody was then applied, and secondary antibodies were subsequently applied for detection. The DAKO Envision Detection System was used for detecting PD-L1 staining. PD-L1 expression was measured with inForm Cell Analysis software (PerkinElmer, Waltham, MA, USA) using the PD-L1 combined positive score (CPS), defined as the number of PD-L1-positive cells (tumour cells, lymphocytes, and macrophages) as a proportion of the total number of tumour cells multiplied by 100. The microsatellite status of each case was assessed with four DNA mismatch repair (MMR) proteins (MLH1, PMS2, MSH2, and MSH6). MMR protein staining was performed with anti-MLH1 antibody (G168-728, BD Biosciences, 1:50 dilution), PMS2 (A16-4, BD Biosciences, 1:100 dilution), and MSH2 (Ab-2, Merck Millipore, Burlington, MA, USA, 1:100 dilution). Deficient MMR (dMMR) was defined as the absence of nuclear staining of one or more of the MMR proteins in the tumour cells.

### Statistical analysis

Comparisons between samples collected before the initial and second administrations of nivolumab and at the time of PD were performed using the Kruskall-Wallis and Wilcoxon signed-rank tests. Progression-free survival (PFS) was defined as the time from registration in this study to the day of disease progression or death from any cause. PFS was evaluated with Kaplan–Meier curves. The multivariate correlation analysis of the proportion of immune cell subsets, the serum cytokine concentrations, and PFS as the clinical outcome was performed using the pairwise comparison method. PFS was compared between subsets using the log-rank test. Hazard ratios (HRs) and 95% confidence intervals (95%CIs) were calculated using a Cox proportional hazards model. Multivariate analysis was also performed with immune cell subsets and the background characteristics of patients including ECOG PS (PS 0–1 or 2), MMR status (dMMR or proficient MMR), and PD-L1 CPS (CPS ≥ 1 or CPS < 1). *p* < 0.05 was considered significant. All statistical analyses were carried out using JMP 13 (SAS Institute Inc., Cary, NC, USA).

## Results

### Patients

**Fig. 1:**
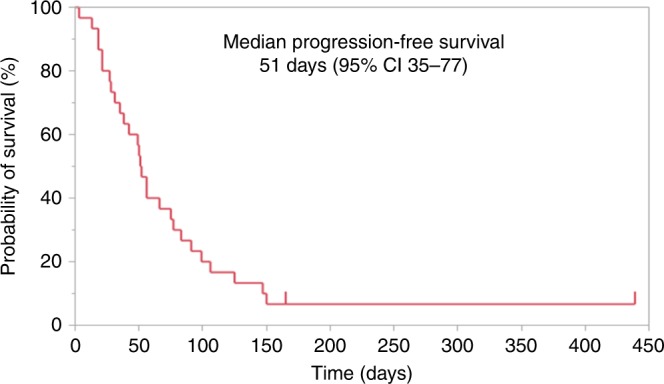
Kaplan–Meier plot of progression-free survival in enrolled patients (*n* = 30). Marks on the curve indicate patients who were censored. The horizontal axis indicates progression-free survival (PFS), and the vertical axis indicates the rate of PFS. PFS was defined as the time since study enrolment to progressive disease (PD) or death from any reason. Median PFS is 51 days (95% CI 35–77 days).

The patients’ baseline characteristics are shown in Table 1. Thirty AGC patients were included in this study between 14 November 2017 and 14 December 2018. Two patients (6.7%) received nivolumab as second-line therapy, and 28 patients (93%) received it as third- or later-line therapy. One patient (3%) was dMMR, and 17 patients (57%) showed PD-L1 CPS of 1 or higher. A summary of treatment response, survival, and reasons for discontinuation is shown in Table 2. Objective response was seen in 20%. Twenty-nine patients (96.7%) discontinued nivolumab therapy, due to PD (28 patients, 96.6%) and the adverse effect of myocarditis (1 patient, 3.4%). The median follow-up period was 140 days (range, 17–504 days). The median PFS was 51 days (95%CI, 35–77 days) by March 2019. The Kaplan–Meier plots of PFS in enrolled patients are shown in Fig. 1. One PR case and one SD case were censored. A severe adverse event associated with nivolumab was observed in one case who developed myocarditis and discontinued nivolumab. Peripheral blood samples at the time of PD could not be obtained from five patients. For the seven patients who were diagnosed with PD by the investigator before the second administration of nivolumab, the second blood sampling was considered the blood sampling at the time of PD. Thus, blood samples were obtained from 30 patients before the initial administration of nivolumab, 23 patients before the second administration, and 25 patients at the time of PD. The data of each time-point sample were compared. The patients’ characteristics at each time-point are shown in Supplementary table 1. The representative immunohistochemical staining of the primary lesions for MMR proteins and PD-L1 is shown in Supplementary Figs. 1 and 2.

**Table 1:** Patients’ baseline characteristics.

Characteristic	*n* = 30
Age	Median (range)
years	69 (36–82)
Sex	*n* (%)
Male	19 (63%)
Female	11 (37%)
ECOG PS	
0	5 (17%)
1	17 (57%)
2	8 (27%)
HER2 status	
positive	7 (23%)
negative	23 (77%)
Previous treatment regimens	
1	2 (6.7%)
2	21 (70%)
3	7 (23%)
Previous therapy	
Fluoropyrimidine	28 (93%)
Platinum	24 (80%)
Taxane	26 (87%)
Irinotecan	3 (10%)
Ramucirumab	22 (73%)
Trastuzumab	5 (17%)
MSI/ MMR status	
dMMR	1 (3%)
PD-L1 CPS	
≥1	17 (57%)
<1	12 (40%)
Unknown	1 (3%)

*Age* median and range, *ECOG PS* Eastern Cooperative Oncology Group performance status, *HER2* human epidermal growth factor receptor 2, *MSI* microsatellite instability, *MMR* DNA mismatch repair, *dMMR* deficient MMR, *CPS* combined positive score.

**Table 2:** Summary of treatment responses and reasons for discontinuation of nivolumab.

Tumor response data	n (%)
Best overall response	
CR	0 (0%)
PR	1 (3.3%)
SD	5 (16.7%)
PD	24 (80%)
Treatment cycle	
Median (range)	3.5 (1–21)
Reason for discontinuation of nivolumab	
PD	28 (96.6%)
Adverse events	1 (3.4%)
Withdrawal of consent	0 (0%)

*CR* complete remission, *PR* partial response, *SD* stable disease, *PD* progressive disease.

### Changes in immune cell subsets after administration of nivolumab

**Fig. 2:**
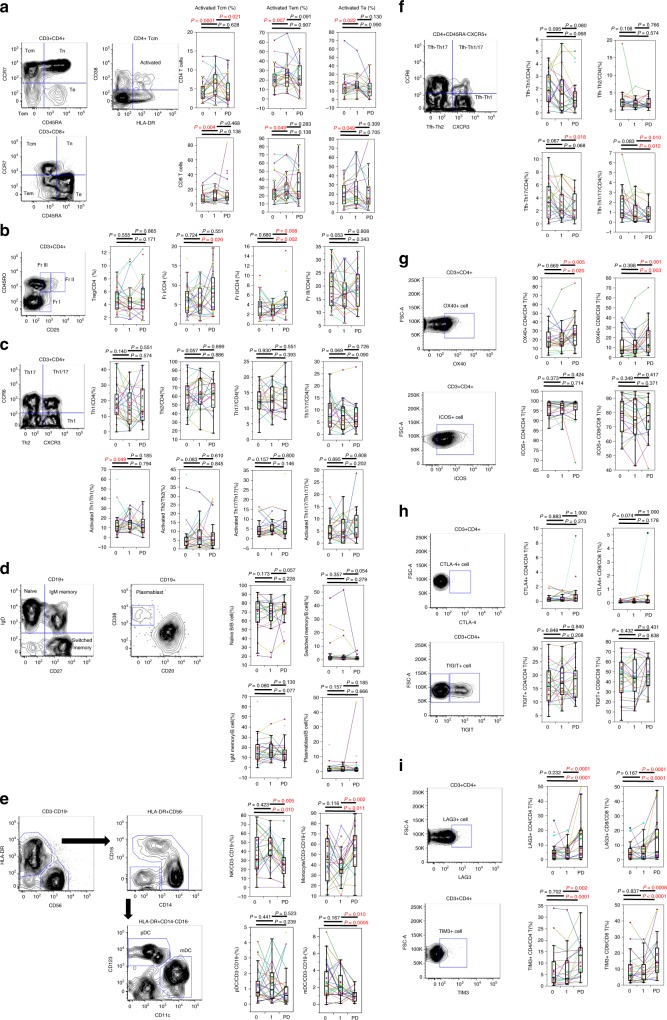
Changes in immune cell phenotypes after anti-programmed death (anti-PD)-1 antibody treatment. The gating strategy and the change in each immune cell phenotype after administration of nivolumab are shown. **a** Proportion of activated CD4+ (CD8+) central/effector memory/effector cells. **b** Proportion of regulatory T cells (Treg) fractions (FrI, FrII, and FrIII) among CD4+ T cells. **c** Proportion of T-helper (Th) cells and of activated Th subsets among CD4+ T cells. **d** Proportion of naïve B cells, IgM memory B cells, switched memory B cells, and plasmablasts among CD19+ B cells. **e** Proportion of natural killer (NK) cells, monocytes, and dendritic cells (DCs) among CD3-CD19- mononuclear cells. **f** Proportion of T-helper follicular (Tfh) cells among CD4+ T cells. **g** Proportion of co-stimulatory marker (OX40, ICOS)-positive cells among CD4+/CD8+ T cells. **h** Proportion of co-inhibitory marker (CTLA-4, TIGIT)-positive cells among CD4+/CD8+ T cells. **i** Proportion of exhaustion marker (LAG3, TIM3)-positive cells among CD4+/CD8+ T cells. Cycle 0, pre-treatment, that is, prior to the first PD-1 antibody cycle; cycle 1, post-first treatment cycle (i.e. prior to the second PD-1 antibody cycle); PD, at the time of progressive disease; Matched patient samples are connected by coloured lines.

The changes in immune cell subsets were evaluated in 30 AGC patients at 3 time points: prior to treatment, after 1 cycle of treatment, and at the time of PD. The changes in the proportions of CD4+ T cells and CD8+ T cells in the CD3+ T cells did not show significant trends. The proportions of naïve T cells (Tn, CD3+CD45RA+CCR7+), central memory T cells (Tcm, CD3+CD45RA−CCR7+), effector memory T cells (Tem, CD3+CD45RA−CCR7−), and effector T cells (Te, CD3+CD45RA+CCR7−) in the CD4+ and CD8+ T cells showed no significant differences after 1 cycle of nivolumab and at the time of PD (data not shown). CD4+ or CD8+ T cells expressing the activated phenotype (CD38+HLA-DR+) increased after administration of nivolumab in the Tcm, Tem, and Te subsets (Fig. 2a). On the other hand, these subsets tended to decrease at the time of PD, but they did not show significant changes except for CD4+Tcm (Fig. 2a). The naïve Treg fraction (FrI, CD3+CD4+CD45RO−CD25dim) and activated Treg fraction (FrII, CD3+CD4+CD45RO+CD25high) increased significantly at the time of PD (Fig. 2b). No significant difference in the proportions of helper T cell (Th) subsets, including Th1 cells (CD45RA-CXCR5-CXCR3+CCR6−), Th2 cells (CD45RA−CXCR5−CXCR3−CCR6−), Th17 cells (CD45RA−CXCR5−CXCR3−CCR6+), and Th1/17 cells (CD45RA−CXCR5−CXCR3+CCR6+), was observed after 1 cycle of treatment (Fig. 2c). The proportion of activated Th1 cells (CXCR3+CCR6−CD38+HLA−DR+) in the Th1 cell population increased significantly after 1 cycle of therapy, but no difference was observed for Th2, Th17, or Th1/17 (Fig. 2c). In terms of B cell subsets, no significant change was observed in the proportion of naïve B cells (CD19+IgD+CD27−), switched memory B cells (CD19+IgD−CD27+), IgM memory B cells (CD19+IgD+CD27+), transitional B cells (CD19+CD24+CD38+), and plasmablasts (CD19+CD20−CD38 high) in CD19+ cells (Fig. 2d). The proportion of NK cells (CD3-CD19-CD56+) and myeloid DCs (mDC, HLA-DR+CD14-CD11c+CD123-) in the CD3-CD19- cell population decreased significantly at the time of PD. On the other hand, the proportion of monocytes (CD3-CD19-HLA-DR+CD14low/+) in the CD3-CD19- cell population increased significantly at the time of PD. No significant change was observed in plasmacytoid DCs (pDCs, HLA-DR+CD14-CD123+) after anti-PD-1 therapy and at the time of PD (Fig. 2e). No significant difference in the proportion of follicular helper T cell (Tfh) subsets, includingTfh-Th1 cells (CD45RA-CXCR5+CXCR3+CCR6-), Tfh-Th2 cells (CD45RA−CXCR5+CXCR3−CCR6-), Tfh-Th17 cells (CD45RA−CXCR5+CXCR3−CCR6+) and Tfh-Th1/17 cells (CD45RA−CXCR5+CXCR3+CCR6+), was observed after 1 cycle of treatment (Fig. 2f). The proportion of Tfh-Th17 cells and Tfh-Th1/Th17 cells in CD4+ T cells decreased significantly at the time of PD (Fig. 2f). In terms of costimulatory (OX40, ICOS) and coinhibitory molecules (CTLA-4, TIGIT) on T cells, the proportion of OX40+ cells in CD4+ and in CD8+ T cells showed a significant increase at the time of PD (Fig. 2g, h). In terms of exhaustion markers (LAG3, TIM3) on T cells, the proportion of both LAG3+ cells and TIM3+ cells in the CD4+ and CD8+ T cells increased significantly (Fig. 2i). Taken together, peripheral blood immune cell profiles after 1 cycle of anti-PD-1 therapy and at the time of PD showed significant increases or decreases as follows: (i) increase in activated CD4+/CD8+ Tcm, Tem and Te, and activated Th1 cells after 1 cycle of therapy; (ii) decrease in NK cells, myeloid DCs, Tfh-Th17, and Tfh-Th1/17 at the time of PD; and (iii) increase in monocytes and CD4+/CD8+OX40+, TIM3+, and LAG3+T cells at the time of PD. After anti-PD-1 treatment, it was technically difficult to detect PD-1 on peripheral blood T cells with flow cytometry. The competitive binding assay of other anti-PD-1 monoclonal antibodies (NAT105, MIH4, and EH12.2H7) with nivolumab targeting PD-1 on peripheral T cell was performed, but no antibody recognised distinctly different epitopes of the PD-1 molecule from nivolumab (data not shown).

### Changes in cytokine concentrations after administration of nivolumab

**Fig. 3:**
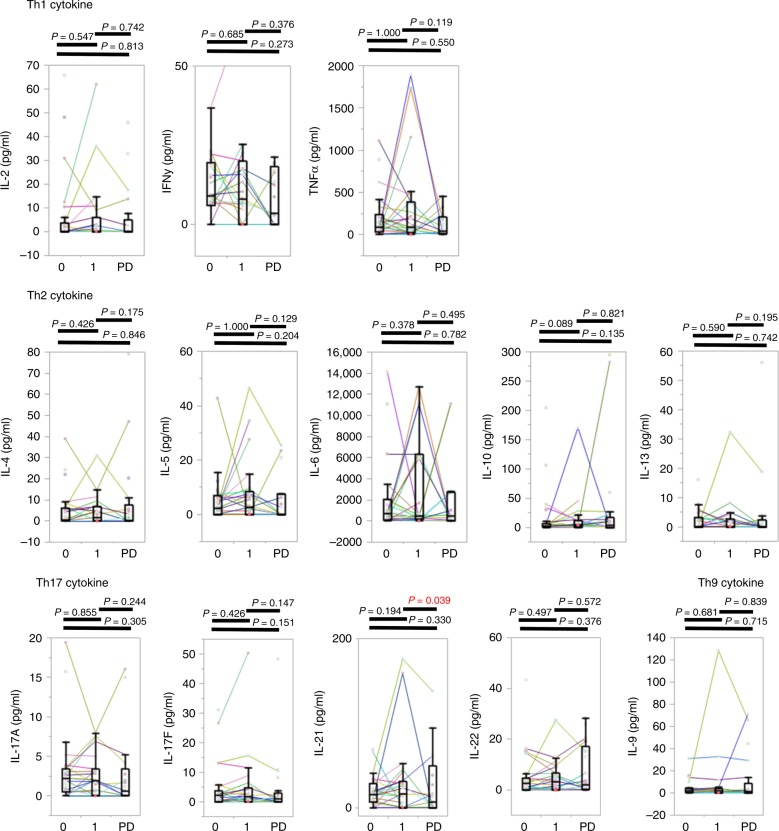
Changes in cytokines after anti-PD-1 antibody treatment. The changes of serum Th cytokine levels (pg/mL) at 3 time points: prior to treatment, after 1 cycle of treatment, and at the time of PD. Cycle 0, cycle 1, and PD in the figure mean the time point prior to first treatment, post-first treatment cycle, and at the time of PD, respectively. Matched patient samples are connected by coloured lines.

The changes in serum Th cytokine concentrations were also evaluated in 30 AGC patients at 3 time points: prior to treatment, after 1 cycle of treatment, and at the time of PD. The serum IL-21 concentration decreased at the time of PD (Fig. 3). The other cytokines did not show significant changes during the time course (Fig. 3).

### Associations between immune cell phenotype/serum cytokine concentration and prognosis

**Fig. 4:**
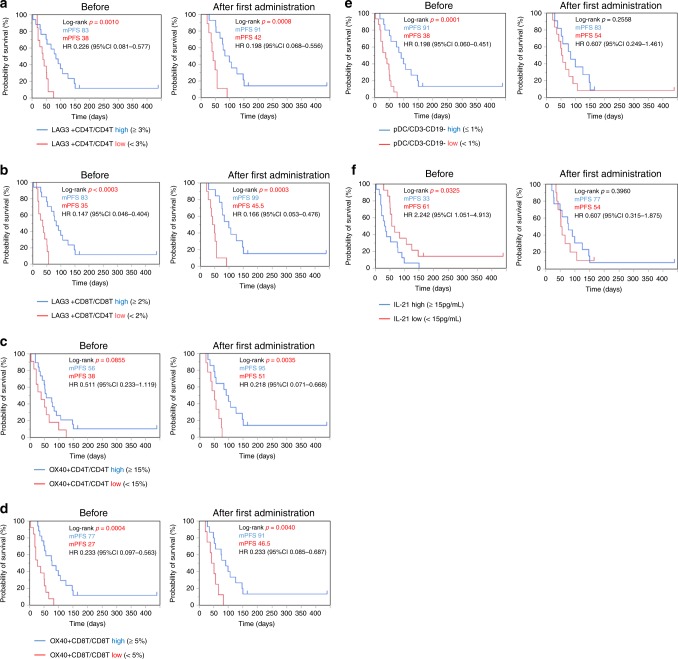
Kaplan–Meier plots of PFS according to immune cell phenotypes and cytokines. PFS by treatment groups in patients according to the ratio of immune cell subsets and serum cytokine concentrations. The median PFS (days) and hazard ratio (HR, 95% CI) of each group are shown. **a** “CD4+LAG3-high” group (CD4+LAG3+ T cells/CD4+ T cells ≥3%, blue curve) and “CD4+LAG3-low” group (CD4+LAG3+ T cells /CD4+ T cells <3% red curve). **b** “CD8+LAG3-high” group (CD8+LAG3+ T cells /CD8+ T cells ≥2%, blue curve) and “CD8+LAG3-low” group (CD8+LAG3+ T cells/CD8+ T cells <2% red curve). **c** “CD4+OX40-high” group (CD4+OX40+ T cells/CD4+ T cells ≥15%, blue curve) and “CD4+OX40-low” group (CD4+OX40+ T cells/CD4+ T cells <15% red curve). **d** “CD8+OX40-high” group (CD8+OX40+ T cells/CD8+ T cells ≥5%, blue curve) and “CD8+OX40-low” group (CD8+OX40+ T cells/CD8+ T cells <5% red curve). **e** “pDC-high” group (pDC/CD3-CD19- cells ≥1%, blue curve) and “pDC-low” group (pDC/CD3-CD19- cells <1% red curve). **f** “IL-21-high” group (serum level of IL-21 ≥ 15 pg/mL, blue curve) and “serum level of IL-21 < 15 pg/mL, red curve).

The multivariate correlations of the proportion of baseline immune cell phenotype (%) and serum cytokine concentration (pg/mL) with median PFS (days) were assessed using a pairwise comparison method. CD4+/CD8+LAG3+ T cells in CD4+/CD8+ T cells, CD4+/CD8+OX40+ T cells in CD4+/CD8+T cells, and pDC/CD3-CD19- cells showed positive correlations with PFS (correlation coefficient: *r* = 0.7321, 0.6343, 0.3807, 0.4695 and 0.3586, *p* ≤ 0.0001, 0.0002, 0.038, 0.0089, 0.033 and 0.0517). On the other hand, IL-21 showed a tendency for a negative correlation with PFS (correlation coefficient: *r* = −0.3409, *p* = 0.0652). Next, the association between each parameter and PFS was evaluated with Kaplan–Meier curves. Patients in whom the proportion of CD4+OX40+ T cells in CD4+ T cells was higher than the median were defined as the “CD4+OX40-high” group, and those with a lower proportion were defined as the “CD4+OX40-low” group. In the same way, patients were categorised into “high” and “low” groups according to the median in terms of the proportion of CD8+OX40+ T cells in CD8+ T cells, CD4+/CD8+LAG3+ T cells in CD4+/CD8+ T cells, and pDCs in CD3-CD19- cells, and the serum concentration of IL-21: “CD8+OX40-high” and “CD8+OX40-low”; and “IL-21-high” and “IL-21-low”. Kaplan–Meier plots were drawn for the “high” and “low” groups. Prior to nivolumab therapy, PFS was significantly longer in the high group than in the low group in terms of CD8+OX40+ T cells, CD4+/CD8+LAG3+ T cells, and pDCs (log-rank *p* = 0.0004, 0.0010, <0.0001 and 0.001). After the first administration of nivolumab and at the time of PD, PFS was significantly longer in the high group than in the low group in terms of CD4+/CD8+OX40+ T cells, CD4+/CD8+LAG3+ T cells (log-rank *p* = 0.0035, 0.0040, 0.0008 and 0.0003). On the other hand, PFS in the high group was significantly shorter than in the low group in terms of IL-21 (log-rank *p* = 0.0325). No significant differences in PFS were observed, however, in terms of pDC after the first therapy (Fig. 4a–f). These results suggest that LAG3+ T cells, OX40+ T cells, and pDCs are associated with a better prognosis. On the other hand, IL-21 was associated with a poorer prognosis. Multivariate analysis (Cox proportional hazard model) with OX40+/LAG3+ high or low groups and the background characteristics of patients including performance status (PS 0-1 or 2), MMR status (dMMR or proficient MMR), and CPS (CPS ≥ 1 or CPS < 1) was also performed. Prior to nivolumab therapy, the hazard ratio (HR) for PFS with the high group versus the low group in terms of CD8+OX40+ T cells and CD4+/CD8+LAG3+ T cells was 0.0837 (95% CI 0.0231-0.3030), 0.2673 (95% CI 0.1017-0.7025), and 0.1526 (95% CI 0.0502–0.4634), respectively (Likelihood ratio test, *p* < 0.0001, 0.0055, 0.0003). After the first administration of nivolumab, the HR for PFS with the high group versus the low group in terms of CD4+/CD8+OX40+ T cells and CD4+/CD8+LAG3+ T cells was 0.2705 (95% CI 0.0876-0.8354), 0.1206 (95% CI 0.0310-0.4693), 0.1164 (95% CI 0.0294-0.4604), and 0.0926 (95% CI 0.0220-0.3900), respectively (Likelihood ratio test, *p* = 0.0189, 0.0021, 0.0013 and 0.0004). At the time of PD, the HR for PFS with the high group versus the low group in terms of CD4+/CD8+OX40+ T cells and CD4+/CD8+LAG3+ T cells was 0.2450 (95% CI 0.0844–0.7110), 0.2289 (95% CI 0.0753–0.6959), 0.3796 (95% CI 0.1333–1.0813), and 0.3095 (95% CI 0.1111–0.8623), respectively (Likelihood ratio test, *p* = 0.0116, 0.0111, 0.0779 and 0.0245). In addition, the phenotype of OX40+/LAG3+ T cells, which is associated with prognosis, was evaluated. OX40+CD4+ T cells showed a significantly higher proportion of Tcm phenotype and a significantly lower proportion of Tn phenotype than OX40-CD4+ T cells (mean ± standard error, 44.1% ± 2.79% vs 20.4% ± 1.62% and 10.7% ± 1.47% vs 35.6% ± 4.32%, Wilcoxon signed-rank test *p* < 0.001 and <0.001), and OX40+CD8+ T cells showed a significantly higher proportion of Tcm phenotype and significantly lower proportion of Tem phenotype than OX40-CD8+ T cells (9.75% ± 1.55% vs 5.50% ± 1.15% and 29.9% ± 3.06% vs 36.7% ± 3.42%, *p* < 0.001 and 0.006). LAG3+CD4+ T cells showed a significantly higher proportion of Tn and significantly lower proportion of Tcm and Tem phenotype than LAG3-CD4+ T cells (60.5% ± 4.78% vs 24.5% ± 3.22%, 14.4% ± 1.73% vs 27.9% ± 2.09% and 13.6% ± 2.46% vs 38.9% ± 3.75%, *p* < 0.001, <0.001 and <0.001). LAG3+CD8+ T cells showed a significantly higher proportion of Tn and Te phenotype and significantly lower proportion of Tcm and Tem phenotype than LAG3-CD8+ T cells (23.0% ± 4.57% vs 5.96% ± 1.22%, 71.8% ± 4.72% vs 25.6 ± 2.67%, 1.06% ± 0.31% vs 12.4% ± 1.79% and 4.08% ± 1.03% vs 56.1% ± 3.13%, *p* < 0.001, <0.001, <0.001 and <0.001). In addition, OX40+ cells showed no significant difference in the proportion of Tregs compared to OX40-T cells (2.24% ± 0.30% vs 2.54% ± 0.39%, *p* = 0.860), and LAG3+ T cells showed a significantly lower proportion of Tregs than that LAG3-T cells (1.23% ± 0.22% vs 2.57% ± 0.32%, *p* < 0.001), suggesting that Tregs are not a dominant population among LAG3/OX40+ T cells. These results are shown in Supplementary Fig. 3.

## Discussion

In this study, peripheral blood immune cells were comprehensively studied to clarify specific subsets that correlate closely with the efficacy of anti-PD-1 therapy for AGC patients. The median PFS of the present cohort was 1.72 months (95% CI: 1.17–2.57 months), which is almost equivalent to that in the ATTRACTION-02 study (1.61 months, 95% CI:1.54–2.30 months),^7^ even though PS 2 patients accounted for 27% of the patients in the cohort. The changes in the proportion of immune cell subsets during the time course of anti-PD-1 therapy showed a similar pattern to our previous findings in malignant melanoma (MM) patients. A significant increase in activated CD4+/CD8+ Tcm, Tem, Te and Th1 after 1 cycle of anti-PD-1 treatment and the trend of their decreases at the time of PD were observed. These observations were almost the same as those in MM cases.^21^ Tn differentiates into Te after antigen presentation by APCs in secondary lymphoid tissues, and some Te survives and exists as Tcm or Tem. Effector and memory T cells are antigen-specific populations after priming.^25,26^ PD-1 blockade not only reinvigorates the cytotoxicity of CD8+ T cells in tumour tissue, but it also enhances tumour antigen priming to Tn in secondary lymphoid tissues. In fact, T cells in lymph nodes are activated, and CD8+ Tcms in tumour infiltrating T cells (TILs) are increased after anti-PD-1 therapy.^26–28^ In non-squamous cell lung cancer patients, the T cell repertoire of peripheral blood before and after nivolumab administration and of TILs in the resected tumour tissue was evaluated, and neo-antigen specific T cell clones appeared and increased transiently after PD-1 therapy.^29^ Peripheral blood T cells exhibiting memory and effector phenotypes in AGC patients may not necessarily possess anti-tumour activity. They might include a non-tumour-specific T cell population, and they might also include exhausted T cells due to continuous tumour antigen presentation. However, the activated memory cells, effector cells, and Th1 cells that increased transiently after anti-PD-1 therapy may contain certain tumour antigen-specific clones. In this study, Treg subsets were classified by surface markers, with the CD3+CD4+CD45RO-CD25-low population as naïve Tregs (Fr I), and the CD3+CD4+CD45RO+CD25-high population as effector Tregs (Fr II).^21^ Naïve Tregs differentiate into effector Tregs, and effector Tregs suppress T cell function.^30^ The proportion of Fr II at the time of PD showed a significant increase, similar to our previous study of MM.^21^ In previous reports of MM and ovarian cancer, the proportion of Tregs in peripheral blood or tumour tissue was correlated with clinical stage,^31,32^ and Tregs of peripheral blood increased in non-responders to anti-PD-1 therapy after treatment, whereas they decreased in responders,^33^ indicating that an increase of Tregs in peripheral blood or tumour tissue may induce a state refractory to anti-PD-1 therapy. In the present study, increases in naïve/effector Tregs were observed at the time of PD, and this effector Treg fraction, which has a T cell inhibitory function, was thought to be functionally enhanced at the time of PD. Effector Tregs may be an effective biomarker for predicting the progression of disease during anti-PD-1 therapy. Exhaustion markers (TIM3, LAG3, TIGIT) were analysed on T cells. PD-1 expression on the T cell surface is induced by T cell activation, and its expression increases with T cell exhaustion. Therefore, it is difficult to definitively identify the status of T cell exhaustion with PD-1 expression alone. The expressions of exhaustion markers, PD-1, TIM3, LAG3, and TIGIT, are correlated with the expression of the transcription factor Eomesodermin (Eomes) in chronic infections and tumour models.^34,35^ Eomes-high PD-1-high T cells, which show deficiencies in T cell functions such as cytokine production, proliferation, and cytotoxicity, have been reported to be difficult to reinvigorate with PD-1 administration.^34–36^ In fact, in cancerous ascites of gastrointestinal cancer patients during the time course of PD, multiple exhaustion markers, PD-1- and TIM3-positive T cells, increased, and this is thought to be a phenomenon that reflects the progression of T cell exhaustion.^37^ The expressions of multiple exhaustion markers were then analysed in the present study. The proportions of LAG3- and TIM3-positive T cells showed no significant changes after anti-PD-1 therapy. However, these subsets increased significantly at the time of PD. T cells expressing LAG3 and TIM3 at the time of PD were thought to be exhausted populations with deficiencies in functions and the inability to respond to anti-PD-1 therapy. A high proportion of OX40+/LAG-3+ T cells before and after the anti-PD-1 treatment was significantly correlated with a better prognosis in the present study. OX40 is a member of the TNFR superfamily, expressed on the surface of activated CD4+/CD8+ T cells, Tregs, and transmits costimulatory signals by binding to OX40 ligand (OX40L).^38,39^ The OX40/OX40L signal is thought to be involved in the formation and survival of memory CD4+/CD8+ T cells^40–42^ and has been reported to suppress inhibitory functions of Tregs and differentiation into Tregs.^43^ Furthermore, OX40 agonistic antibody enhanced infiltration of CD8+ T cells into tumour tissues and cytotoxicity against tumour cells,^44,45^ suggesting its contribution to the anti-tumour effects of OX40/OX40L signalling. A higher proportion of OX40-positive T cells has been observed in peripheral blood of chronic graft-versus-host disease cases and gastric cancer cases,^46,47^ but there has been no report of the correlation with prognosis in solid tumours, to the best of our knowledge. In addition, it has been reported that OX40 expression of TILs and prognosis were positively correlated in colorectal cancer and MM cases.^48,49^ These findings suggested that OX40-associated formation and survival of memory T cells and suppression of Tregs might enhance the anti-tumour effect and contribute to the favourable prognosis. Neo-antigen-specific T cell clones have emerged and increased transiently after anti-PD-1 therapy,^29^ which may enhance anti-tumour effects by OX40/OX40L signalling. On the other hand, LAG3 binds to MHC class II and negatively regulates T cell activation, cytotoxicity, and cytokine production. LAG3 is expressed on activated CD4+/CD8+ T cells and Tregs and is overexpressed with the exhaustion of T cells.^50^ Although LAG3 is the exhaustion marker, LAG3 expression on the cell surface first requires T cell activation.^51^ A positive correlation has been observed between LAG3 expression of TILs and prognosis in oesophageal cancer, non-small cell lung cancer, and microsatellite instability-high colorectal cancer cases.^52–54^ The expression of PD-1 and LAG-3 in TILs is positively correlated,^55^ and it has been reported that inhibition of both PD-1 and LAG3 showed a synergistic effect in T cell activation.^56^ In addition, CD8+ T cells expressing PD-1, TIM3 and LAG3 were thought to be tumour-reactive and neo-antigen-specific.^57^ Taken together, LAG3-positive T cells express PD-1, and PD-1/PD-L1 blockade might restore the anti-tumour effect of tumour-specific exhausted T cells, which might lead to improvement of the prognosis. In the present study, LAG3-positive T cells, which are associated with a better prognosis after anti-PD-1 therapy, might have included an exhausted subset that is tumour-specific and can be reinvigorated by PD-1/PD-L1 blockade. On the other hand, LAG3-positive T cells at the time of PD might express multiple exhaustion markers and Eomes and be too exhausted to respond to anti-PD-1 therapy.

The predictive factors for response to anti-PD-1 antibody in gastric cancer have been reported: PS, MSI/dMMR status, and PD-L1 CPS.^58,59^ However, other predictive factors are not well known. In the present study, the proportion of OX40+/LAG3+ T cells was found to be a potential predictive factor independent of these background factors on multivariate analysis (Cox proportional hazard model).

This study has several limitations. First, this was a prospective study, but it was based on actual clinical practice and included various patient backgrounds, and CT was performed by the attending physicians based on the clinical practice; thus, the CT evaluation period was not clearly defined. The fact that the CT evaluation was not subject to independent central imaging facility review was also considered to be a limitation in this study. However, this study was based on actual clinical practice and considered to be valuable in that respect. Second, the number of antigens that could be analysed by flow cytometry at one time was limited due to the technical limitation of fluorescence leakage, and 10 panels of fluorophore-conjugated monoclonal antibodies were used for immunophenotyping. Thus, there was difficulty in evaluating the status of co-expression of some antigens. One of the future prospects is multidimensional analysis of protein expression at the single-cell level using a new platform such as mass cytometry.

Activated Tcm, Tem, and Te increased after anti-PD-1 therapy in AGC patients. These proportions of OX40 and LAG3-positive T cells in peripheral blood before and after anti-PD-1 therapy are expected to be predictive biomarkers for anti-PD-1 therapy. Further analyses of the changes of immune cell subsets with anti-PD-1 therapy are required to better understand the immunological and clinical impact of PD-1-blockade in gastric cancer patients.

## Supplementary information

Supplementary table, figures and figure legends

## Data Availability

The data used and analysed during this study are available from the corresponding author on reasonable request.

## References

[1] GLOBOCAN 2018, Data visualization tools for exploring the global cancer burden in 2018. http://gco.iarc.fr/today/fact-sheets-cancer (2019)

[2] Ajani JA, D’Amico TA, Almhanna K, Bentrem DJ, Chao J, Das P (2016). Gastric Cancer, Version 3.2016, NCCN clinical practice guidelines in oncology. J. Natl Compr. Canc. Netw..

[3] Hironaka S, Ueda S, Yasui H, Nishina T, Tsuda M, Tsumura T (2013). Randomized, open-label, phase III study comparing irinotecan with paclitaxel in patients with advanced gastric cancer without severe peritoneal metastasis after failure of prior combination chemotherapy using fluoropyrimidine plus platinum: WJOG 4007 trial. J. Clin. Oncol..

[4] Thuss-Patience PC, Kretzschmar A, Bichev D, Deist T, Hinke A, Breithaupt K (2011). Survival advantage for irinotecan versus best supportive care as second-line chemotherapy in gastric cancer-a randomised phase III study of the Arbeitsgemeinschaft Internistische Onkologie (AIO). Eur. J. Cancer.

[5] Fuchs CS, Tomasek J, Yong CJ, Dumitru F, Passalacqua R, Goswami C (2014). Ramucirumab monotherapy for previously treated advanced gastric or gastro-oesophageal junction adenocarcinoma (REGARD): an international, randomised, multicentre, placebo-controlled, phase 3 trial. Lancet.

[6] Wilke H, Muro K, Van Cutsem E, Oh SC, Bodoky G, Shimada Y (2014). Ramucirumab plus paclitaxel versus placebo plus paclitaxel in patients with previously treated advanced gastric or gastro-oesophageal junction adenocarcinoma (RAINBOW): a double-blind, randomised phase 3 trial. Lancet Oncol..

[7] Kang YK, Boku N, Satoh T, Ryu MH, Chao Y, Kato K (2017). Nivolumab in patients with advanced gastric or gastro-oesophageal junction cancer refractory to, or intolerant of, at least two previous chemotherapy regimens (ONO-4538-12, ATTRACTION-2): a randomised, double-blind, placebo-controlled, phase 3 trial. Lancet.

[8] Zhang X, Schwartz JC, Guo X, Bhatia S, Cao E, Lorenz M (2004). Structural and functional analysis of the costimulatory receptor programmed death-1. Immunity.

[9] Hsu J, Hodgins JJ, Marathe M, Nicolai CJ, Bourgeois-Daigneault MC, Trevino TN (2018). Contribution of NK cells to immunotherapy mediated by PD-1/PD-L1 blockade. J. Clin. Invest.

[10] Yi JS, Cox MA, Zajac AJ (2010). T-cell exhaustion: characteristics, causes and conversion. Immunology.

[11] Chen DS, Mellman I (2013). Oncology meets immunology: the cancer-immunity cycle. Immunity.

[12] Weber JS, D’Angelo SP, Minor D, Hodi FS, Gutzmer R, Neyns B (2015). Nivolumab versus chemotherapy in patients with advanced melanoma who progressed after anti-CTLA-4 treatment (CheckMate 037): a randomised, controlled, open-label, phase 3 trial. Lancet Oncol..

[13] Brahmer J, Reckamp KL, Baas P, Crino L, Eberhardt WE, Poddubskaya E (2015). Nivolumab versus Docetaxel in Advanced Squamous-Cell Non-Small-Cell Lung Cancer. N. Engl. J. Med..

[14] Horn L, Spigel DR, Vokes EE, Holgado E, Ready N, Steins M (2017). Nivolumab Versus Docetaxel in Previously Treated Patients With Advanced Non-Small-Cell Lung Cancer: Two-Year Outcomes From Two Randomized, Open-Label, Phase III Trials (CheckMate 017 and CheckMate 057). J. Clin. Oncol..

[15] Motzer RJ, Escudier B, McDermott DF, George S, Hammers HJ, Srinivas S (2015). Nivolumab versus Everolimus in Advanced Renal-Cell Carcinoma. N. Engl. J. Med..

[16] Ansell SM, Lesokhin AM, Borrello I, Halwani A, Scott EC, Gutierrez M (2015). PD-1 blockade with nivolumab in relapsed or refractory Hodgkin’s lymphoma. N. Engl. J. Med..

[17] Ferris RL, Blumenschein G, Fayette J, Guigay J, Colevas AD, Licitra L (2016). Nivolumab for Recurrent Squamous-Cell Carcinoma of the Head and Neck. N. Engl. J. Med..

[18] Okazaki T, Honjo T (2007). PD-1 and PD-1 ligands: from discovery to clinical application. Int. Immunol..

[19] Pardoll D, Drake C (2012). Immunotherapy earns its spot in the ranks of cancer therapy. J. Exp. Med..

[20] Maecker HT, McCoy JP, Nussenblatt R (2012). Standardizing immunophenotyping for the Human Immunology Project. Nat. Rev. Immunol..

[21] Yamaguchi K, Mishima K, Ohmura H, Hanamura F, Ito M, Nakano M (2018). Activation of central/effector memory T cells and T-helper 1 polarization in malignant melanoma patients treated with anti-programmed death-1 antibody. Cancer Sci..

[22] Ohmura, H., Yamaguchi, K., Hanamura, F., Ito, M., Makiyama, A., Uchino, K., et al. Activation of central/effector memory T cells in advanced gastric cancer patients treated with antiprogrammed death-1 antibody. *J Clin Oncol*. **37(Suppl 4)**, (abstract 54) (2019).

[23] Eisenhauer EA, Therasse P, Bogaerts J, Schwartz LH, Sargent D, Ford R (2009). New response evaluation criteria in solid tumours: revised RECIST guideline (version 1.1). Eur. J. Cancer.

[24] National Cancer Institute. Common Terminology Criteria for Adverse Events (CTCAE) version 4.0. 2010. http://evs.nci.nih.gov/ftp1/CTCAE/CTCAE_4.03_2010-06 14_QuickReference_8.5x11.pdf (2017)

[25] MacLeod MK, Kappler JW, Marrack P (2010). Memory CD4 T cells: generation, reactivation and re-assignment. Immunology.

[26] Salmon H, Idoyaga J, Rahman A, Leboeuf M, Remark R, Jordan S (2016). Expansion and activation of CD103(+) dendritic cell progenitors at the tumor site enhances tumor responses to therapeutic PD-L1 and BRAF inhibition. Immunity.

[27] Goldberg MV, Maris CH, Hipkiss EL, Flies AS, Zhen L, Tuder RM (2007). Role of PD-1 and its ligand, B7-H1, in early fate decisions of CD8 T cells. Blood.

[28] Ribas A, Shin DS, Zaretsky J, Frederiksen J, Cornish A, Avramis E (2016). PD-1 blockade expands intratumoral memory T cells. Cancer Immunol. Res..

[29] Forde PM, Chaft JE, Pardoll DM (2018). Neoadjuvant PD-1 blockade in resectable lung cancer. N. Engl. J. Med..

[30] Miyara M, Yoshioka Y, Kitoh A, Shima T, Wing K, Niwa A (2009). Functional delineation and differentiation dynamics of human CD4+ T cells expressing the FoxP3 transcription factor. Immunity.

[31] Gambichler T, Bindsteiner M, Hoxtermann S, Terras S, Kreuter A (2013). Circulating CD4+ CD25(high) CD127(low) regulatory T cells are an independent predictor of advanced melanoma. Pigment Cell Melanoma Res..

[32] Leffers N, Gooden MJ, de Jong RA, Hoogeboom BN, ten Hoor KA, Hollema H (2009). Prognostic significance of tumor-infiltrating T-lymphocytes in primary and metastatic lesions of advanced stage ovarian cancer. Cancer Immunol. Immunother..

[33] Weber JS, Kudchadkar RR, Yu B, Gallenstein D, Horak CE, Inzunza HD (2013). Safety, efficacy, and biomarkers of nivolumab with vaccine in ipilimumab-refractory or -naive melanoma. J. Clin. Oncol..

[34] Chen DS, Mellman I (2017). Elements of cancer immunity and the cancer-immune set point. Nature.

[35] Jia B, Zhao C, Rakszawski KL, Claxton DF, Ehmann WC, Rybka WB (2019). Eomes(+)T-bet(low) CD8(+) T cells are functionally impaired and are associated with poor clinical outcome in patients with acute myeloid leukemia. Cancer Res.

[36] Li J, He Y, Hao J, Ni L, Dong C (2018). High levels of eomes promote exhaustion of anti-tumor CD8(+) T cells. Front. Immunol..

[37] Nakano M, Ito M, Tanaka R, Yamaguchi K, Ariyama H, Mitsugi K (2018). PD-1+ TIM-3+ T cells in malignant ascites predict prognosis of gastrointestinal cancer. Cancer Sci..

[38] Curti BD, Kovacsovics-Bankowski M, Morris N, Walker E, Chisholm L, Floyd K (2013). OX40 is a potent immune-stimulating target in late-stage cancer patients. Cancer Res..

[39] Croft M (2003). Co-stimulatory members of the TNFR family: keys to effective T-cell immunity?. Nat. Rev. Immunol..

[40] Bansal-Pakala P, Halteman BS, Cheng MH, Croft M (2004). Costimulation of CD8 T cell responses by OX40. J. Immunol..

[41] Mousavi SF, Soroosh P, Takahashi T, Yoshikai Y, Shen H, Lefrancois L (2008). OX40 costimulatory signals potentiate the memory commitment of effector CD8+ T cells. J. Immunol..

[42] Soroosh P, Ine S, Sugamura K, Ishii N (2007). Differential requirements for OX40 signals on generation of effector and central memory CD4+ T cells. J. Immunol..

[43] Xiao X, Kroemer A, Gao W, Ishii N, Demirci G, Li XC (2008). OX40/OX40L costimulation affects induction of Foxp3+ regulatory T cells in part by expanding memory T cells in vivo. J. Immunol..

[44] Gough MJ, Ruby CE, Redmond WL, Dhungel B, Brown A, Weinberg AD (2008). OX40 agonist therapy enhances CD8 infiltration and decreases immune suppression in the tumor. Cancer Res..

[45] Peng W, Williams LJ, Xu C, Melendez B, McKenzie JA, Chen Y (2019). Anti-OX40 antibody directly enhances the function of tumor-reactive CD8(+) T cells and synergizes with PI3Kbeta inhibition in PTEN loss melanoma. Clin. Cancer Res..

[46] Martins MR, Santos RLD, Jatahy KDN, Matta MCD, Batista TP, Junior JIC (2018). Could OX40 agonist antibody promote activation of the anti-tumor immune response in gastric cancer?. J. Surg. Oncol..

[47] Kotani A, Ishikawa T, Matsumura Y, Ichinohe T, Ohno H, Hori T (2001). Correlation of peripheral blood OX40+(CD134+) T cells with chronic graft-versus-host disease in patients who underwent allogeneic hematopoietic stem cell transplantation. Blood.

[48] Petty JK, He K, Corless CL, Vetto JT, Weinberg AD (2002). Survival in human colorectal cancer correlates with expression of the T-cell costimulatory molecule OX-40 (CD134). Am. J. Surg..

[49] Ladanyi A, Somlai B, Gilde K, Fejos Z, Gaudi I, Timar J (2004). T-cell activation marker expression on tumor-infiltrating lymphocytes as prognostic factor in cutaneous malignant melanoma. Clin. Cancer Res..

[50] Long L, Zhang X, Chen F, Pan Q, Phiphatwatchara P, Zeng Y (2018). The promising immune checkpoint LAG-3: from tumor microenvironment to cancer immunotherapy. Genes Cancer.

[51] Bae J, Lee SJ, Park CG, Lee YS, Chun T (2014). Trafficking of LAG-3 to the surface on activated T cells via its cytoplasmic domain and protein kinase C signaling. J. Immunol..

[52] Hald SM, Rakaee M, Martinez I, Richardsen E, Al-Saad S, Paulsen EE (2018). LAG-3 in non-small-cell lung cancer: expression in primary tumors and metastatic lymph nodes is associated with improved survival. Clin. Lung Cancer.

[53] Lee SJ, Jun SY, Lee IH, Kang BW, Park SY, Kim HJ (2018). CD274, LAG3, and IDO1 expressions in tumor-infiltrating immune cells as prognostic biomarker for patients with MSI-high colon cancer. J. Cancer Res. Clin. Oncol..

[54] Zhang Y, Liu YD, Luo YL, Liu BL, Huang QT, Wang F (2018). Prognostic value of lymphocyte activation Gene-3 (LAG-3) expression in esophageal squamous cell carcinoma. J. Cancer.

[55] He Y, Yu H, Rozeboom L, Rivard CJ, Ellison K, Dziadziuszko R (2017). LAG-3 protein expression in non-small cell lung cancer and its relationship with PD-1/PD-L1 and tumor-infiltrating lymphocytes. J. Thorac. Oncol..

[56] Lichtenegger FS, Rothe M, Schnorfeil FM, Deiser K, Krupka C, Augsberger C (2018). Targeting LAG-3 and PD-1 to enhance T cell activation by antigen-presenting cells. Front. Immunol..

[57] Gros A, Robbins PF, Yao X, Li YF, Turcotte S, Tran E (2014). PD-1 identifies the patient-specific CD8(+) tumor-reactive repertoire infiltrating human tumors. J. Clin. Invest.

[58] Shitara K, Ozguroglu M, Bang YJ, Di Bartolomeo M, Mandala M, Ryu MH (2018). Pembrolizumab versus paclitaxel for previously treated, advanced gastric or gastro-oesophageal junction cancer (KEYNOTE-061): a randomised, open-label, controlled, phase 3 trial. Lancet.

[59] Marabelle A, Le DT, Ascierto PA, Di Giacomo AM, De Jesus-Acosta A, Delord JP (2020). Efficacy of Pembrolizumab in patients with noncolorectal high microsatellite instability/mismatch repair-deficient cancer: results from the phase II KEYNOTE-158 Study. J. Clin. Oncol..

