# Supplementary material for: OX40 and LAG3 are associated with better prognosis in advanced gastric cancer patients treated with anti-programmed death-1 antibody
Source: Br J Cancer. 2020 Mar 23;122(10):1507–17. doi: 10.1038/s41416-020-0810-1 (PMC7217874; doi:10.1038/s41416-020-0810-1)
Supplement: Supplementary file 1 — Supplementary table, figures and figure legends [file 41416_2020_810_MOESM1_ESM.pdf]

Supplementary table 1. Patients' baseline characteristics during the time course of therapy

Characteristic	Before nivolumab therapy n=30	After nivolumab therapy n=23	At the time of PD n=25
**Age**	**Median (range)**		
years	69 (36 - 82)	69 (36 - 82)	68 (36 - 80)
**Sex**	**n (%)**		
Male	19 (63%)	15 (65%)	17 (63%)
Female	11 (37%)	8 (35%)	8 (37%)
**ECOG PS**			
0	5 (17%)	5 (22%)	4 (16%)
1	17 (57%)	17 (74%)	15 (60%)
2	8 (27%)	8 (35%)	6 (24%)
**HER2 status**			
positive	7 (23%)	6 (23%)	5 (23%)
negative	23 (77%)	17 (77%)	20 (77%)
**Previous treatment regimens**			
1	2 (6.7%)	2 (8.7%)	2 (8%)
2	21 (70%)	15 (65%)	17 (68%)
3	7 (23%)	6 (26%)	6 (24%)
**Previous therapy**			
Fluoropyrimidine	28 (93%)	21 (91%)	23 (92%)
Platinum	24 (80%)	18 (78%)	19 (76%)
Taxane	26 (87%)	20 (87%)	22 (88%)
Irinotecan	3 (10%)	21 (93%)	23 (92%)
Ramucirumab	22 (73%)	18 (78%)	17 (68%)
Trastuzumab	5 (17%)	4 (17%)	4 (16%)
**MSI/ MMR status**			
dMMR	1 (3%)	1 (5%)	0 (0%)
**PD-L1 CPS**			
≥1	17 (57%)	12 (52%)	14 (56%)
<1	12 (40%)	10 (43%)	10 (40%)
Unknown	1 (3%)	1 (5%)	1 (4%)

Age (median and range), ECOG PS (Eastern Cooperative Oncology Group performance status), HER2 (Human epidermal growth factor receptor 2), MSI (Microsatellite instability), MMR (DNA Mismatch repair), dMMR (deficient MMR), CPS (Combined positive score)

Supplementary Figure 1

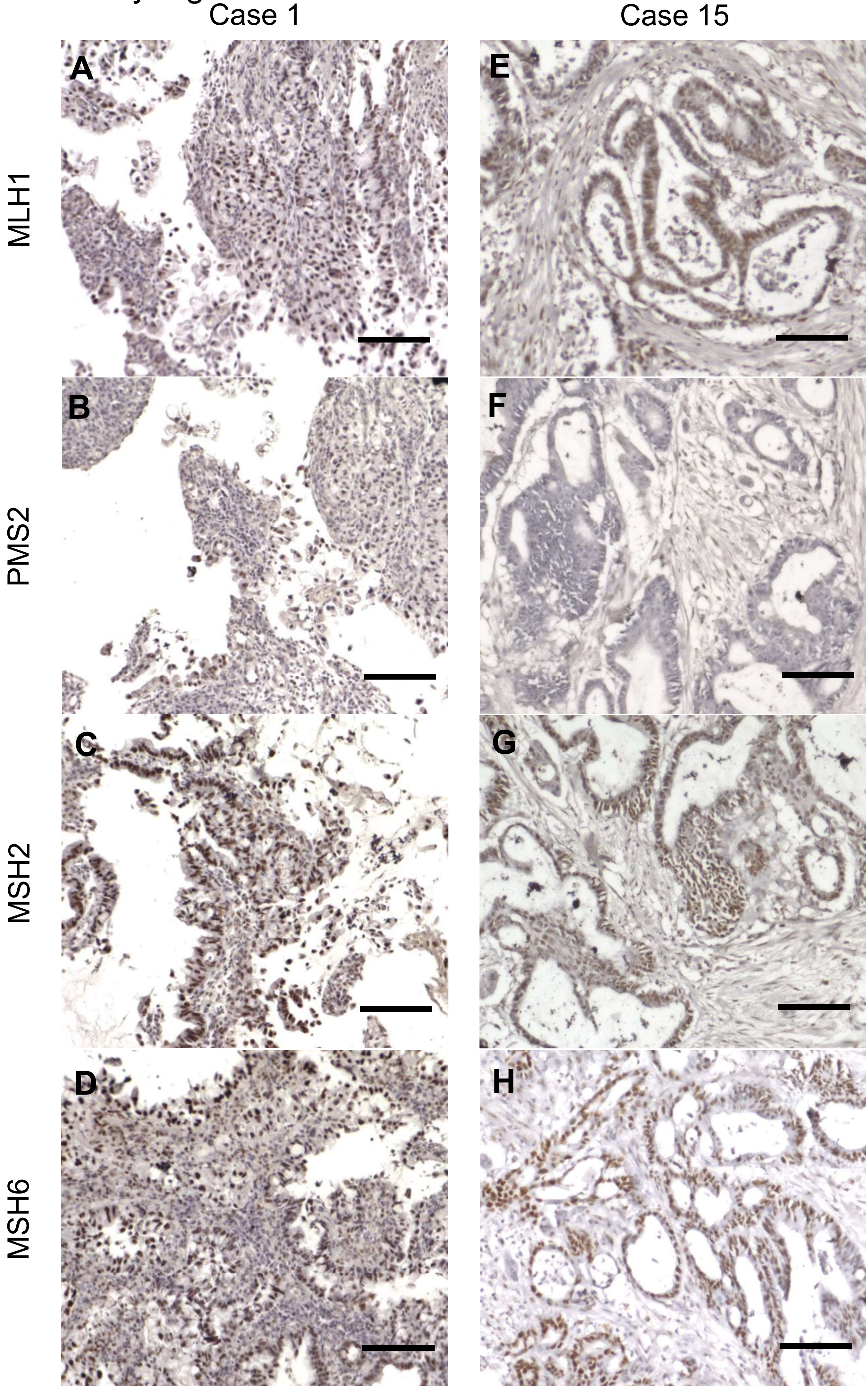

Supplementary Figure 2

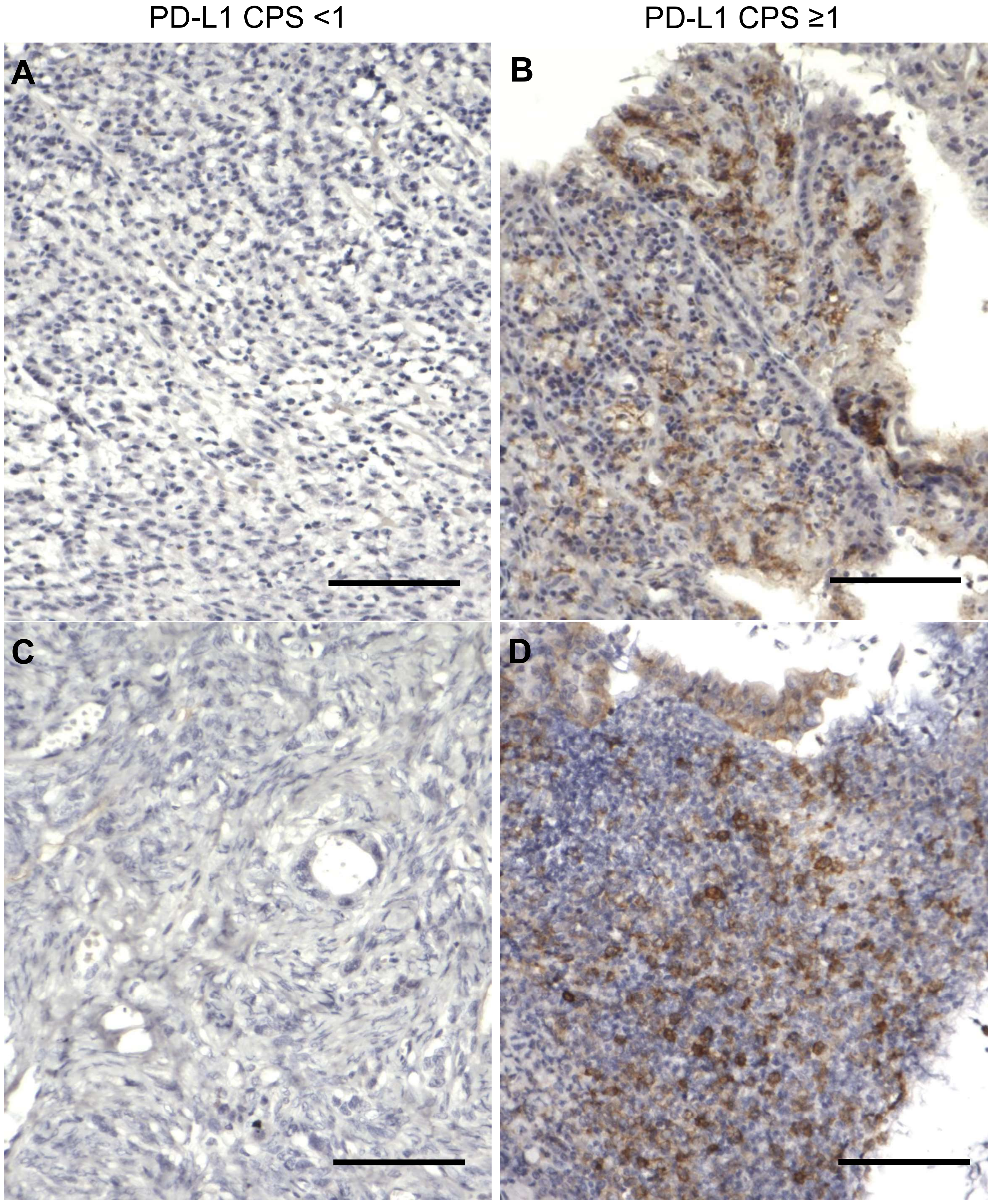

Supplementary Figure 3

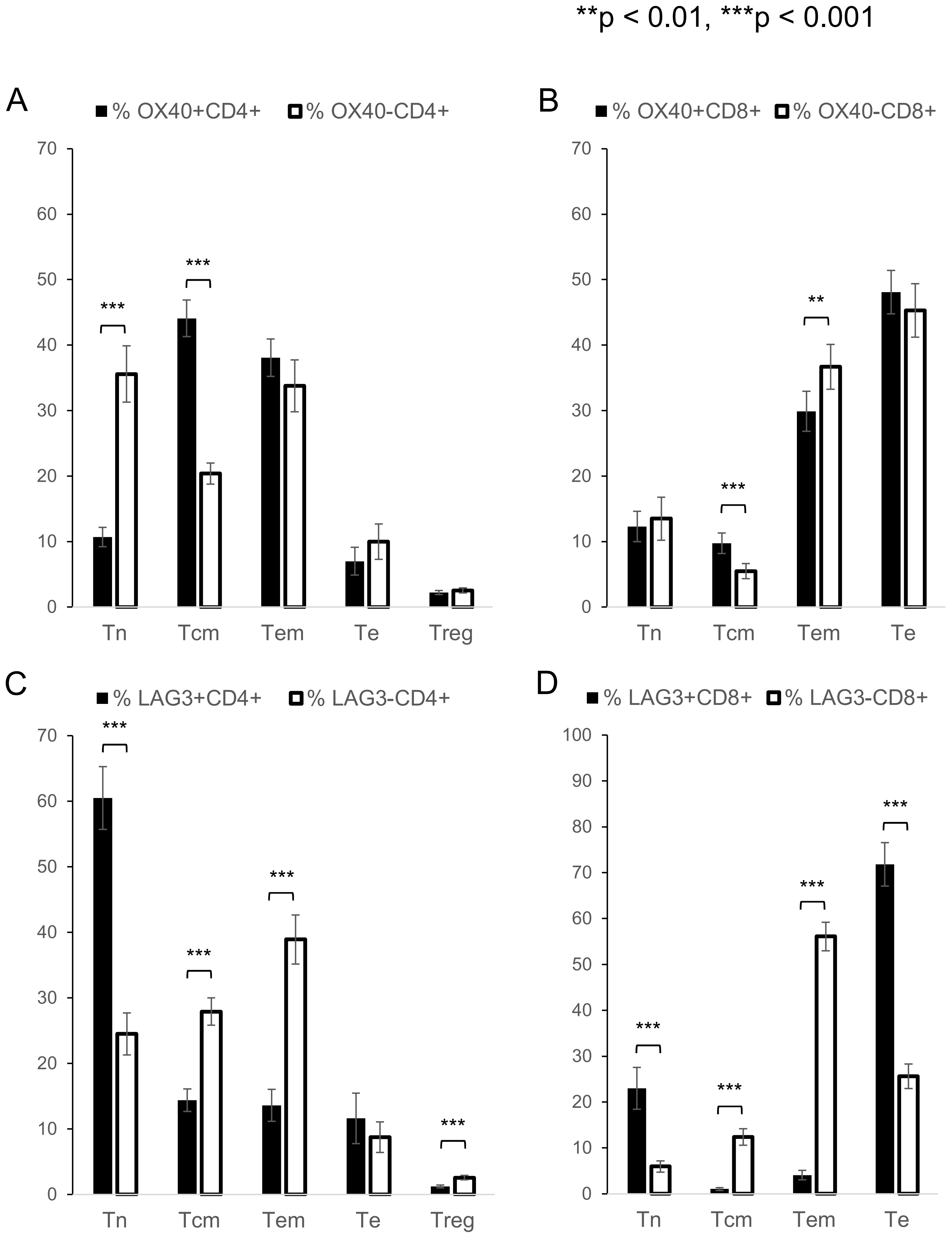

SUPPLEMENTARY FIGURE LEGENDS

Supplementary figure 1: Immunohistochemical staining of primary lesions for mismatch repair (MMR) proteins. **A-D**, A representative MMR-proficient case (case 1) shows nuclear staining of all four MMR proteins in tumor tissue (scale bar 100 μm). **E-H**, The MMR-deficient case (case 15) shows loss of PMS2 protein in tumor cells (scale bar 100 μm).

Supplementary figure 2: Immunohistochemical staining of primary lesions for PD-L1. **A, C**, A representative PD-L1 combined positive score (CPS) <1 case (case 10 and case 30). **B, D**, A representative PD-L1 CPS ≥1 case (case 11 and case 27) (scale bar 100 μm).

Supplementary figure 3: A, The proportions of naïve T cells (Tn), central memory T cells (Tcm), effector memory T cells (Tem), effector T cells (Te), and regulatory T cells (Treg) in OX40+/OX40-CD4+T cells are shown. Columns, means of 23 patients; black columns mean OX40+CD4+T cells, and white columns mean OX40-CD4+T cells; bars, SEM; **p <0.01; ***p <0.001. **B**, The proportions of Tn, Tcm, Tem, and Te in OX40+/OX40-CD8+T cells. **C**, The proportions of Tn, Tcm, Tem, Te, and Tregs in LAG3+/LAG3-CD4+T cells. **D**, The proportions of Tn, Tcm, Tem, and Te in LAG3+/LAG3-CD8+T cells.
